# From pancreatic ductal adenocarcinoma to non-small cell lung cancer: daraxonrasib in the RASolute 302 trial – a pan-RAS(ON) breakthrough or the next evolution in targeting diverse oncogenic RAS variants?

**DOI:** 10.1097/MS9.0000000000005188

**Published:** 2026-05-21

**Authors:** Muhammad Qaseem, Mian Sajjad Ali, Shehbaz Ali, Abdul Haseeb Hasan

**Affiliations:** a Department of Medicine, Khyber Medical College, Peshawar, Pakistan; b Department of Research, Oli Health Magazine Organization, Kigali, Rwanda; c Department of Internal Medicine, Sentara Northern Virginia Medical Center, Woodbridge, Virginia, USA; d Department of Medicine, King Edward Medical University, Lahore, Pakistan

**Keywords:** daraxonrasib, non-small cell lung cancer, NSCLC, pancreatic ductal adenocarcinoma, PDAC, RAS

*To the Editor,*

Oncogenic RAS-mutated tumors are a range of aggressive solid tumors, most notably pancreatic ductal adenocarcinoma (PDAC) and RAS-mutant non-small cell lung cancer (NSCLC), caused by hotspot mutations in RAS GTPases. Pathogenesis is mainly mutation-based, with G12, G13, or Q61 changes stabilizing the GTP-bound RAS(ON) form and resulting in constitutive MAPK/ERK and PI3K/AKT signaling, leading to proliferation, invasion, and metastasis. These tumors are clinically characterized by advanced stages, rapid progression, and site-specific symptoms. Hotspot RAS mutations are detected in 92% of patients with PDAC, indicating a huge global burden of morbidity and mortality. The epochal RASolute 302 outcomes are a forerunner of a new age of pan-RAS(ON) multiselected therapies. Daraxonrasib, cytotoxic chemotherapy, and selective KRAS G12C inhibitors are also crucial options[1].

RMC-6236 (daraxonrasib) is an oral, noncovalent, tri-complex RAS(ON) multi-selective inhibitor that targets the active GTP-bound state of both mutant and wild-type canonical RAS isoforms (KRAS, HRAS, and NRAS). To some extent, it acts as a molecular glue by binding cyclophilin A with high affinity (Kp1 = 55.3 nmol/L), forming a binary complex that then engages RAS(ON) (Kp2 ranging from 131 to 364 nmol/L), sterically blocking effector interactions such as RAF and potently disrupting RAS/MAPK signaling (EC₅₀ 28–220 nmol/L for RAS–RAF disruption). *In vitro*, it achieved deep growth inhibition (EC₅₀ 1.2–1.4 nmol/L) and sustained pERK suppression in KRAS G12-mutant PDAC cell lines (e.g., HPAC, Capan-2), with a strong correlation to biochemical potency (*r*^2^ = 0.94). *In vivo*, daily oral dosing at 25 mg/kg drove profound tumor regressions in KRAS G12X xenograft models (ORR 52% in NSCLC and 64% in PDAC) and significantly extended progression-free survival, including complete RECIST responses in heavily pretreated KRAS G12D PDAC and KRAS G12V NSCLC patients at 300 mg daily in Phase 1 (NCT05379985). It was well-tolerated with a favorable therapeutic window over normal tissues and showed initial clinical activity in many cases. This supports its advancement primarily in RAS-mutant PDAC (>90% KRAS-driven) and RAS-mutant NSCLC (~30% of cases)[2].

Daraxonrasib (RMC-6236), a pan-RAS(ON) multi-selective noncovalent tri-complex inhibitor, is based on robust preclinical and translational evidence demonstrating broad activity against diverse oncogenic RAS variants in PDAC and NSCLC models. Single oral doses of daraxonrasib (3–25 mg/kg) achieved profound, dose-dependent suppression of RAS signaling (DUSP6 mRNA inhibition up to 98% at 25 mg/kg), with sustained pathway suppression for 48 hours at the highest dose, translating into significant dose-dependent tumor growth inhibition. In pharmacodynamic and efficacy studies using PDAC xenograft models (MIA PaCa-2 KRASG12C and HPAF-II KRASG12D/WT), complementing these findings, optimized lead compounds (including daraxonrasib) showed potent cellular antiproliferative activity across multiple RAS-mutant lines, such as Capan-1 KRASG12V (CTG EC_50_ = 1.3 nM), NCI-H358 KRASG12C (CTG EC_50_ = 1.0 nM), and AsPC-1 KRASG12D (CTG EC_50_ = 3.1 nM), alongside strong disruption of RAS-BRAF RBD interactions (IC50 values as low as 35 nM for G12C), supporting its advancement to Phase 3 trials (RASolute 302 in metastatic PDAC and RASolute 301 in RAS-mutant NSCLC)[3]. In support of these clinical findings, an analysis of post-progression cfDNA from 143 patients treated with first-generation KRAS^G12C^ inhibitors identified acquired RAS/MAPK alterations in 46% of cases. This included KRAS second-hit mutations in 39% and amplifications in 22%, underscoring the limitations of mutant-selective approaches and emphasizing the need for multi-selective strategies, such as RAS(ON) tri-complex inhibitors[4].

Despite the promising initial activity of daraxonrasib (RMC-6236), a pan-RAS(ON) multiselected tri-complex inhibitor, several therapy-specific real-world challenges currently hinder its optimal use in RAS-mutant PDAC and NSCLC. To some extent, G13X and Q61X necessitate advanced NGS or ctDNA testing infrastructure, which remains unavailable or inconsistent outside specialized academic centers. The need for comprehensive molecular profiling to identify diverse oncogenic RAS variants (e.g., G12X causing diarrhea, nausea, and mucosal inflammation) demands specialized clinical expertise and supportive care protocols that are not uniformly accessible in community oncology settings. From a practical standpoint, managing on-target toxicities, such as rash resulting from the inhibition of both mutant and wild-type RAS, is crucial, especially for broader indications and extended treatment durations. The substantial development and manufacturing costs of these intricate tri-complex molecules raise concerns about their long-term affordability and reimbursement. Furthermore, evolving regulatory pathways for companion diagnostics and expanded approvals across diverse RAS variants create accessibility barriers in several healthcare systems. Addressing these practical challenges is essential to translate daraxonrasib’s potential into equitable real-world benefits[5].

Daraxonrasib represents a major breakthrough in the strategy for RAS oncogene targeting across multiple types of cancers, as it combines broad isoform specificity with sustained pathway inhibition and remarkable efficacy in pancreatic and lung cancers resistant to extensive previous treatment. The ability to circumvent resistance developed against mutation-specific inhibitors places it as a critical component of future RAS-targeting drugs. Overcoming issues associated with diagnosis, dealing with toxicity, and accessibility will be crucial to realizing its full therapeutic potential on a global scale.

## Data Availability

Data sharing is not applicable to this article, as no datasets were generated or analyzed during the current study.
